# Personal

**Published:** 1895-03

**Authors:** 


### Personal.

Dr. A. H. Wright, of Toronto, on Tuesday, January, 22, 1895, delivered the third lecture of the series on the history of medicine at the Toronto University. Dr. Wright's subject was Alexandrian Medicine and the conclusions he arrived at were that anatomy, both human and comparative, botany and chemistry were well studied; that every assistance was given to those engaged in original research; that disease was subject to natural laws and necessitated close clinical observation for its cure; that treatment should be made of prime importance; and that the physician should recognize a high conception of his status and duty.

Drs. John D. S. and William E. B. Davis, of Birmingham, Ala., have built a commodious infirmary, which they occupied early in February, 1895. The *Medical Age* says:

> These gentlemen are to be congratulated for their successful work in their chosen specialty. By persistent effort, coupled with their splendid abilities, they have succeeded in establishing for themselves, in the city of Birmingham, a lucrative practice, as well as a reputation in the medical profession.

We cheerfully join in the sentiments expressed so gracefully by the *Age*.

Dr. Albert Vander Veer, of Albany, was elected a regent of the University of the State of New York by the legislature, February 13, 1895. The medical profession has in Dr. Vander Veer a suitable representative on the board of regents of the university, and the state at large, as well as the profession of medicine, is to be congratulated upon his election.

---

Dr. Daniel Lewis, of New York City, has been appointed a member of the state board of health. At a meeting of the New York Academy of Medicine held February 7, 1895, Dr. Lewis was also elected one of the vice-presidents to fill the vacancy caused by the election of Dr. Joseph D. Bryant as president of the academy.

These are deserved recognitions of the professional and social standing of one of New York's able physicians.

---

Dr. A. L. Benedict, of Buffalo, was awarded the Merritt H. Cash prize by the medical society of the State of New York at its meeting held in Albany February 5, 6, 7, 1895. There were three essays in competition. The subject of the successful one was Auscultatory Percussion and Allied Methods of Physical Diagnosis. The award was $100 in money.

---

Dr. H. W. Rogers, of Cleveland, has been elected professor of principles and practice of medicine and clinical medicine in the medical department of the University of Wooster. Dr. Rogers is an alumnus of Buffalo University medical college and was a pupil of Professor Miner during his student years.

---

Dr. Roswell Park, professor of surgery in the Buffalo University medical college, was elected president of the medical society of the State of New York at its recent annual meeting. Dr. Park is also a member of the New York State medical association.

---

Dr. E. E. Blaauw, formerly first assistant at the ophthalmological university clinics, Amsterdam, offers his services as a specialist for eye, ear and throat diseases. He is located at 285 Franklin street, Buffalo. Hours 9–1 and 3–4.

---

Dr. R. T. Rolph, formerly of Fredonia, N. Y., has removed to Dunkirk, where he is permanently located for the active practice of his profession.

